# Characterization of Organics Consistent with β-Chitin Preserved in the Late Eocene Cuttlefish *Mississaepia mississippiensis*

**DOI:** 10.1371/journal.pone.0028195

**Published:** 2011-11-23

**Authors:** Patricia G. Weaver, Larisa A. Doguzhaeva, Daniel R. Lawver, R. Christopher Tacker, Charles N. Ciampaglio, Jon M. Crate, Wenxia Zheng

**Affiliations:** 1 Research and Collections, North Carolina Museum of Natural Sciences, Raleigh, North Carolina, United States of America; 2 Department of Palaeozoology, Swedish Museum of Natural History, Stockholm, Sweden; 3 Department of Earth Sciences, Montana State University, Bozeman, Montana, United States of America; 4 Earth and Environmental Sciences, Wright State University, Celina, Ohio, United States of America; 5 Analytical Chemistry, FAI Materials Testing Lab Incorporated, Marietta, Georgia, United States of America; 6 Department of Marine, Earth and Atmospheric Sciences, North Carolina State University, Raleigh, North Carolina, United States of America; Paleontological Institute of Russian Academy of Science, United States of America

## Abstract

**Background:**

Preservation of original organic components in fossils across geological time is controversial, but the potential such molecules have for elucidating evolutionary processes and phylogenetic relationships is invaluable. Chitin is one such molecule. Ancient chitin has been recovered from both terrestrial and marine arthropods, but prior to this study had not been recovered from fossil marine mollusks.

**Methodology/Principal Findings:**

Organics consistent with β-chitin are recovered in cuttlebones of *Mississaepia mississippiensis* from the Late Eocene (34.36 million years ago) marine clays of Hinds County, Mississippi, USA. These organics were determined and characterized through comparisons with extant taxa using Scanning Electron Microscopy/Energy Dispersive Spectrometry (SEM/EDS), Field Emission Scanning Electron Microscopy (Hyperprobe), Fourier Transmission Infrared Spectroscopy (FTIR) and Immunohistochemistry (IHC).

**Conclusions/Significance:**

Our study presents the first evidence for organics consistent with chitin from an ancient marine mollusk and discusses how these organics have been degraded over time. As mechanisms for their preservation, we propose that the inorganic/organic lamination of the cuttlebone, combined with a suboxic depositional environment with available free Fe^2+^ ions, inhibited microbial or enzymatic degradation.

## Introduction

Chitin is a complex amino-polysaccharide produced by many living organisms, including arthropods, mollusks, nematodes, algae and fungi. It is, after cellulose, the second most abundant biopolymer found in nature [1]. Chitin is a linear polymer consisting mainly of β-(1→4)-linked 2-acetamido-2-deoxy-β-d-glucopyranose units and partially of β-(1→4)-linked 2-amino-2-deoxy-β-d-glucopyranose [2]. In nature, chitin occurs in three polymorphs, either α or β-allomorphs or in γ-form [2], distinguished by differences in the types of chemical linkage employed or by the orientation of the constituent micro-fibrils relative to these linkages [1].

Based primarily on morphological comparisons with extant forms and not on chemical analyses of fossils, chitin is hypothesized to have been a major structural component of invertebrates since the Cambrian, but it may have originated sometime in the Proterozoic [3]. Detection of chitin in fossils is not frequent. There are reports of fossil chitin in pogonophora, and in insect wings from amber [4]. Chitin has also been reported from beetles preserved in an Oligocene lacustrine deposit of Enspel, Germany [5] and chitin-protein signatures have been found in cuticles of Pennsylvanian scorpions and Silurian eurypterids [6].

**Figure 1:**
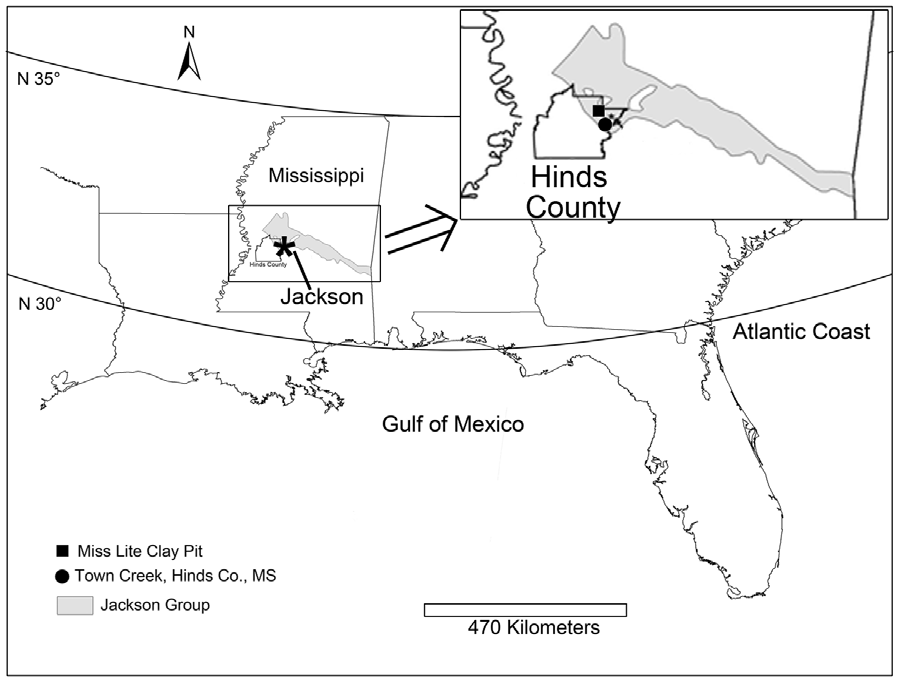
Generalized locality map. Generalized locality map showing sites where *M*. *mississipiensis* were collected and the geographical extent of the Jackson Group in Mississippi.

Initial examination of cuttlebones of *Mississaepia mississippiensis* [7] from the Late Eocene (34.36mya) [8] from the Yazoo Clay, Jackson Group, Hinds County, Mississippi (Figure 1) by light microscopy revealed yellowish-brown sheet-like structures that appeared to have been originally organic. EDS examination of these structures revealed phosphorus. These observations led to questions 1) could any original organics be preserved in these cuttlebones? 2) What mechanisms might have preserved original endogenous organics? The present paper reports evidence of organics consistent with β-chitin from the Late Eocene cephalopod *M*. *mississippiensis* and presents evidence of ultrastructural and environmental factors which might have contributed to their preservation.

## Materials and Methods

**Figure 2:**
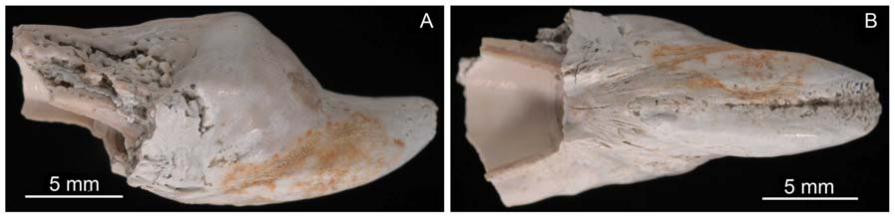
Fragmentary preserved cuttlebone of *M*. *mississippiensis* (MGS 1951). Fragmentary preserved cuttlebone of *M*. *mississippiensis* (MGS 1951), late Eocene, Mississippi, USA: A, B – left lateral and ventral view, respectively.

Nine cuttlebones of *M*. *mississippiensis* from collections at The Mississippi Geological Survey were examined. For comparison, five cuttlebones of extant cuttlefish (*Sepia* sp. NCSM 11399–11403; collections at the North Carolina Museum of Natural Sciences) were examined. Eight fossil specimens (MGS 1943, 1944, 1946–1949, 1956, 1963) and one extant specimen were analyzed using SEM/EDS (Text S1). Specimen MGS 1951 (Figure 2A,B) and extant *Sepia* specimen NCSM 11399 were cut laterally and longitudinally for ground sections. The remaining portion of fossil specimen MGS 1951 and extant specimens NCSM 11402 and 11403 were de-mineralized for Hyperprobe, FTIR and IHC analyses (Text S1). For comparison, three specimens of extant *Loligo* sp. pens were de-mineralized for FTIR analysis. Complete methods are given in the supporting data (Text S1).

## Results

### Scanning Electron Microscopy/Energy Dispersive Spectrometry (SEM/EDS)

**Figure 3:**
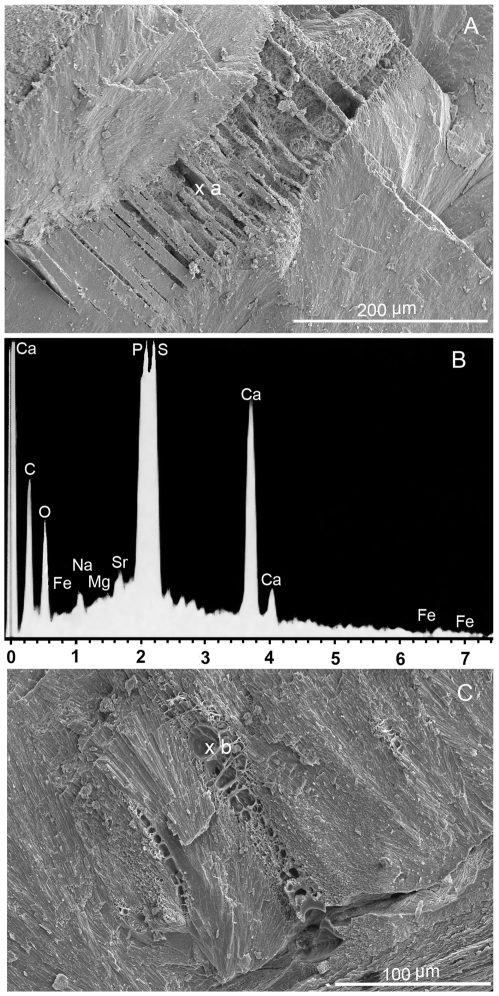
SEM/EDS images comparing similar structures of *M*. *mississippiensis* and *Sepia*. Sheet-like structures in between carbonate spherulites in *M*. *mississippiensis* (MGS1956, Fig. 2Aa) and *Sepia* sp. (Fig. 2Cb). 2B, EDS analysis of *M*. *mississipiensis* (spot a) showing phosophorus in the sheet-like structures.

Analysis of *M*. *mississippiensis* under SEM (Text S1) revealed ultrastructures (Figure 3Aa), between carbonate spherulites, morphologically similar to the chitin membranes found in extant *Sepia* (Figure 3Cb). Elemental analyses show these “sheet-like” structures in *M*. *mississippiensis* contain high amounts of phosphorous (Figure 3B). These sheet-like structures may have been phosphatized during fossilization as is typical of chitin structures in cephalopods, such as gladii and mandibles [9], [10]. Alternatively these originally organic structures could have served as a template for phosphatization [11] and some of the original organic material may have been preserved.

### Hyperprobe

**Figure 4:**
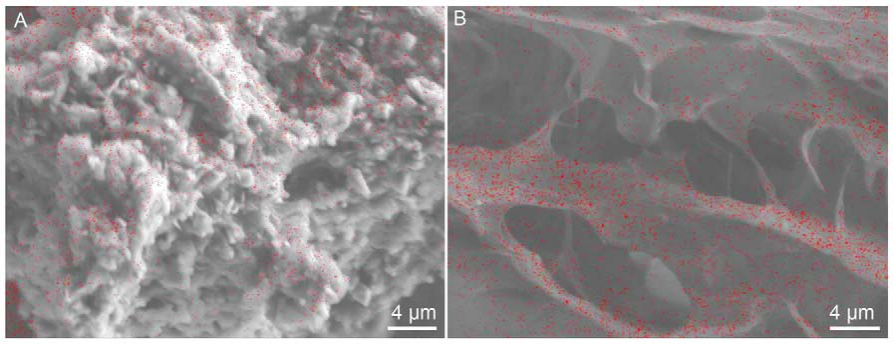
Hyperprobe comparison of HCL de-mineralized *M*. *mississippiensis* and extant *Sepia* sp. A) Hyperprobe analysis of HCL de-mineralized fossil (MGS 1951) mapped for nitrogen (red). B) Hyperprobe analysis of HCL de-mineralized extant cuttlebone mapped for nitrogen (red).

To test for presence of original organics, cuttlebones of *M*. *mississipiensis* and *Sepia* sp. were demineralized in HCL (Text S1) then mapped for nitrogen. Nitrogen, a reliable indicator of organic material [12], was detected in de-mineralized powdered samples of *Mississaepia mississippiensis* and extant *Sepia* sp. using a JEOL X-ray Analyzer JXA-85-30F Hyperprobe (Text S1). Though not a specific indicator of chitin, nitrogen signals obtained from powders indicate original organics have been preserved in the fossil. Nitrogen was detected only on places where there was sample and thus is not a byproduct of nitrogen within the epoxy of the carbon tape (Figure 4A,B). Because these samples are three dimensional no quantitative analyses are possible. Overall morphology of de-mineralized material from *M*. *mississippiensis* and extant *Sepia* sp. was different (Figure 4A,B).

### Fourier Transform Infrared (FTIR)

**Figure 5:**
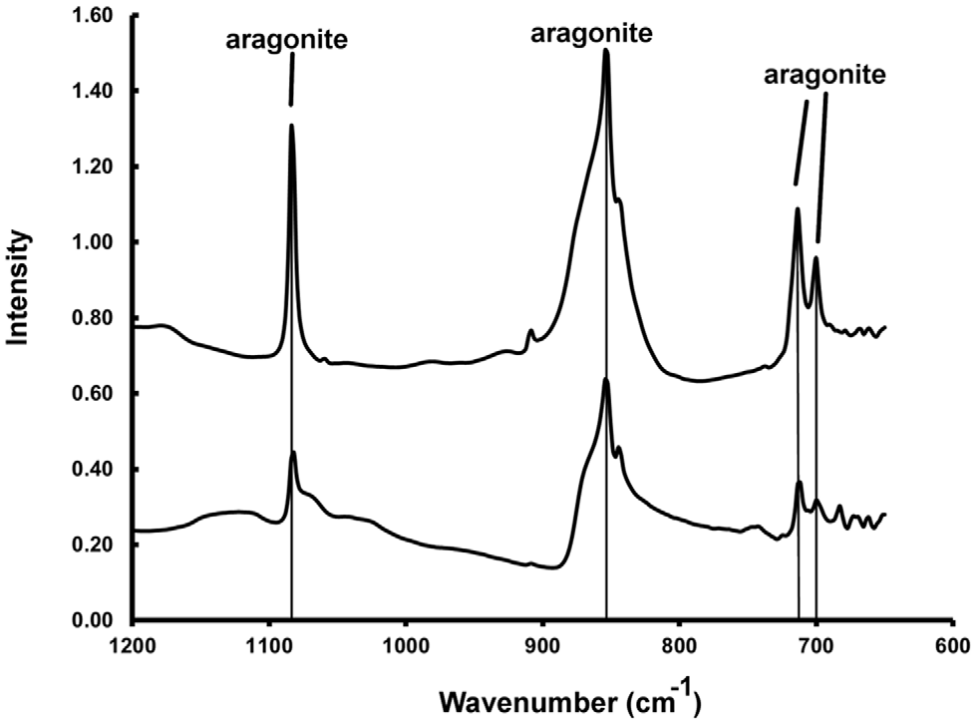
FTIR spectra of cuttlebones of *Sepia* sp. and *M*. *mississippiensis*. Spectra of *Sepia* sp. (top) and *M*. *mississippiensis* (bottom). Spectra indicate aragonite. By comparison, calcite has peaks at 848 and 876 cm^−1^and a singlet at 713 cm^−1^ [14].

Cuttlebones of *M*. *mississippiensis* and *Sepia* sp. were analyzed using FTIR (Text S1) to determine the mineral phase of the carbonates. The cuttlebone of *M*. *mississippiensis* shows two peaks of the carbonate ν_4_ vibration at 700 and 713 cm^−1^ (Figure 5) which are diagnostic of aragonite [13]. The peaks at 1083 and 854 cm^−1^ are also characteristic of aragonite [14]. Similar peaks were detected in cuttlebone of extant *Sepia* (Figure 5). In contrast calcite would show a peak at 876 cm^−1^ and no peak at 1083 cm^−1^ [14]. Preservation of aragonite, a meta-stable form of calcium carbonate, rather than the more stable calcite form, indicate the cuttlebones have undergone minimal diagenesis and thus had a greater potential to preserve original organics.

**Figure 6:**
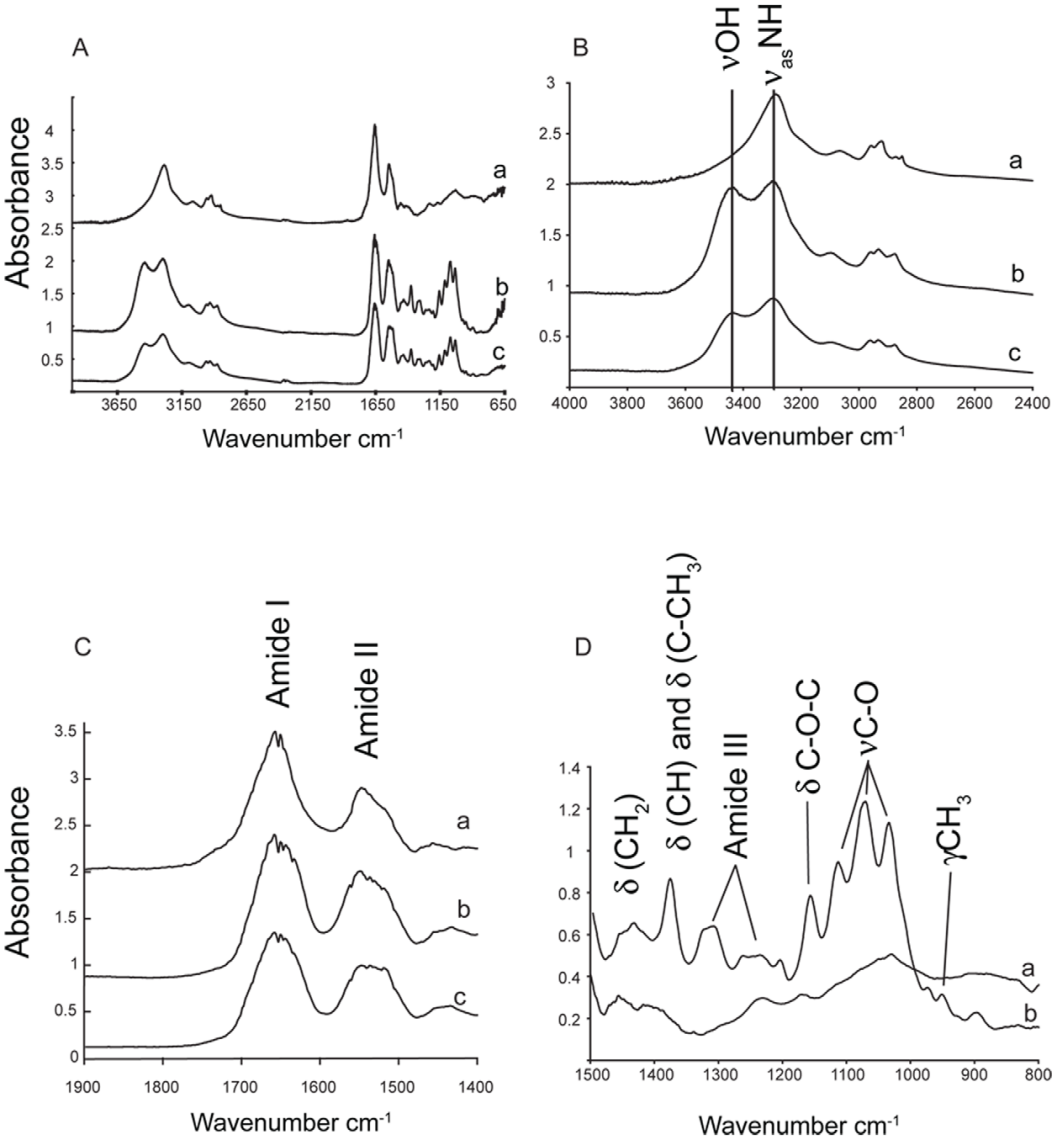
FTIR spectra of HCL de-mineralized *M*. *mississippiensis*, *Sepia* sp. and *Loligo* sp. FTIR spectra of fossil (a) *M*. *mississippiensis* and chitin from extant (b) *Sepia* sp. and (c) *Loligo* sp. A) All spectra are consistent with beta chitin. B) FTIR spectra of the hydrogen stretching region of a, b and c showing reduction in OH of *M*. *mississippiensis*. C) FTIR spectra of a, b and c emphasizing Amide I and Amide II absorbances which are distinctive for beta chitin. D) FTIR spectra of a and b; portions of the Amide III region are missing in the fossil. Overall broadening and loss of structure of the peaks is interpreted as breakdown of chitin chains into shorter fragments.

FTIR (Text S1) was also used to examine in detail the organic components preserved in *M*. *mississippiensis*. After demineralization of extant *Loligo* sp. pens (squid pens are commonly used as the standard for β-chitin), *Sepia* sp. and fossil *M*. *mississippiensis*, spectra (Figure 6A) were obtained and band assignments (Table 1) were made according to published works [13], [15]. Spectra of organics obtained for fossil and extant specimens were similar (Table 1, Figure 6A), though some peaks were not as well resolved in the fossil. The OH stretching absorbance (Figure 6B) was examined using spectral decomposition analysis with Peakfit®. The OH stretching mode in the fossil is greatly reduced as compared to the extant cuttlebone, and shifted to slightly lower wavenumbers (Table 2). The N-H asymmetric stretching vibration is also shifted to slightly lower wavenumbers.

**Table 1:** Band assignments (cm^−1^) for fossil and extant materials.

Band assignment [13], [15]	Squid [15]	*Loligo* sp. (NCSM, Sea of Japan)	*Sepia officinalis* [13]	*Sepia* sp. (NCSM 11403 & 11402)	*M. mississipiensis* MGS 1951
νOH	3479 (?) & 3426	3431	3421	3431	–
ν_as_NH	3290	3292	3280	3395	3281
ν_s_NH	3102	3085	3104	3091	3065
ν_as_CH_3_	2962	2961	2966	2959	2956
ν_s_CH_2_	2929	2933	2933	2931	2918
ν_as_CH_3_	2880	2876	2884	2873	2849
νC = O,Amide I	1656	1656	1652	1657 & 1649	1656 & 1650
νC-N,C-N-H, Amide II +δN-H Amide II	1556	1533	1553	1541	1545
δCH2	1424	1433	1422	1437	1456, 1417
δCH and δC-CH3	1376	1375	1377	1376	1397
νC-N and δNH,Amide III	1314	1324 & 1304	1317	1306	–
δNH, Amide III	1262	1260	1262,1229, 1205	1260,1236, 1204	1231
ν_as_ C-O-C ring	1155	1157	1155	1159	1171
νC-O	1111	1113	1112	1115	–
νC-O	1069	1071	1073	1074	–
νC-O	1032	1033	1033	1032	1030
γCH_3_	948	949	948	947	–
γCH (C1axial) (β bond)	902	895	–	895	905
ρCH_2_	–	–	–	705	703
γNH (Amide V)	692	690	–	695	691

**Table 2:** Peak positions (cm^−1^) from spectral decomposition using Peakfit®.

Band Assignment	*Loligo* sp.	*Sepia* sp.	*M*_._ *mississippiensis*
ν_as_NH	3296	3285	3290
νOH	3444	3446	3405

Peaks at 1649 cm^−1^ (Amide I, Figure 6C) and 1544 cm^−1^(Amide II, Figure 6C) in cuttlefish, squid and fossil spectra indicate organics consistent with β-chitin. Closer examination of the region ca. 600–1400 cm^−1^(Figure 6D) shows the principle change in *M*. *mississippiensis* is the loss of peaks in Amide III region. Amide I and II peaks may arise from chitin, but also from proteins, humics or other chemicals. It is parsimonious to assume the presence of chitin-like molecules rather than an admixture of substances displaying a similar spectrum.

### Immunohistochemistry (IHC)

**Figure 7:**
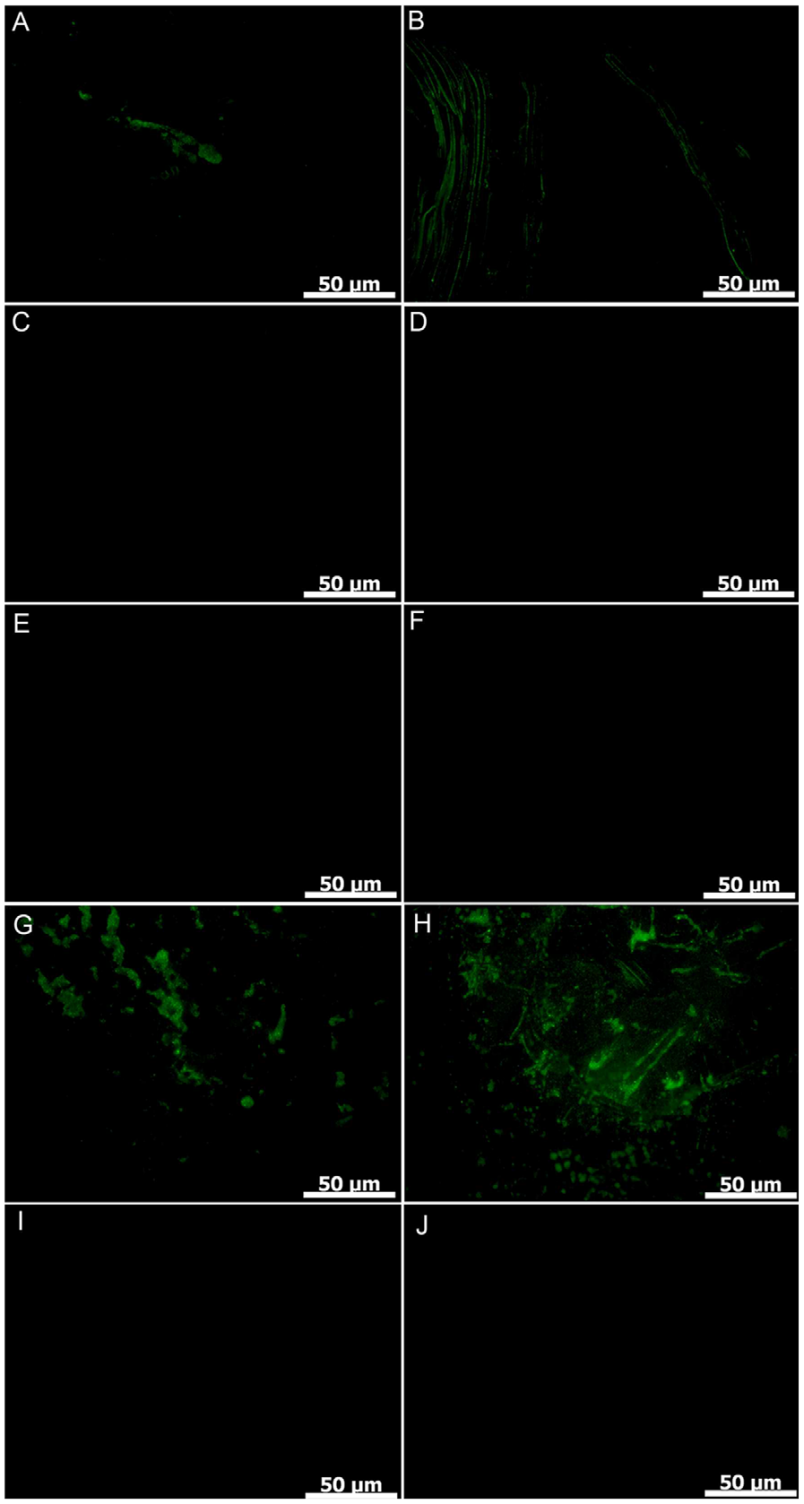
IHC comparison of HCL de-mineralized *M*. *mississippiensis* and *Sepia* sp. A,C,E,G,I, fossil *M*. *mississipiensis* (MGS 1951); B,D,F,H,J, extant *Sepia* sp. A, B) Fossil/extant incubated in chitin antibody and secondary antibody, show antibody/antigen binding. C,D) Fossil/extant incubated in secondary antibody only, no antibody/antigen binding. E,F) Fossil/extant incubated in chitinase, chitin anti-body and secondary antibody. Tissues have been digested by the chitinase giving a much reduced immunological signal. G,H) Fossil/extant incubated in chitinase, chitin antibody and secondary antibody. Tissues in these samples were not completely digested by the chitinase, eptiopes were unmasked, and show enhanced antibody/antigen retrieval. I,J) Fossil/extant incubated in human testosterone antibody and secondary antibody, shows no antibody/antigen binding.

IHC has been used successfully to detect and characterize ancient biomolecules such as collagen in birds [16] and dinosaurs [17]. Because chitin antibodies and IHC have been used to successfully detect chitin in extant organisms [18], [19], IHC (Text S1) was used here to further elucidate the specific nature of biomolecules found in *M*. *mississippiensis*. Analysis of extant *Sepia* sp. and fossil *M*. *mississippiensis* showed specific antibody binding to chitin with comparable avidity and specificity (Figure 7A,B) indicating some endogenous chitin is retained in the fossil, though its morphology is slightly different from chitin in extant *Sepia* sp. (Figure 7A,B). Fluorescent signal from antibody-antigen interaction was greater than that seen in negative controls (secondary antibody only; Figure 7C,D). When sections were subjected to digestion by chitinase, immunological signal was either reduced where tissues were completely digested (Figure 6E,F), or enhanced where tissues were not completely digested (Figure 7G,H). This signal enhancement seems contradictory, but enzymes are often used to help unmask antigens [20]–[22] resulting in an enhanced immunological signal. Degradation by chitinase is indicated by the lack of order (as seen in Figure 7A,B). The pattern of enhanced signal is common to both fossil and extant specimens, indicating commonality in chemical make-up. An irrelevant antibody (human testosterone) was applied at identical concentrations and under identical conditions to some sections to verify specificity of the antibody-antigen interaction. Results showed no antigen/antibody binding (Figure 7I,J). IHC analysis showed fluorescence consistent with and specific to chitin in both fossil and extant samples which is consistent with the interpretation that original chitin was preserved in these specimens.

## Discussion

The initial analysis of fossil *M*. *mississippiensis* by multiple methods yielded results that, taken alone, were insufficient to support the presence of a specific organic material. High percentage of phosphorus detected in EDS analysis shows possible preservation of organic material between aragonitic spherulites. Hyperprobe reveals nitrogen clearly unassociated with carbonate minerals, though the overall morphology of de-mineralized *M*. *mississippiensis* looks different from extant *Sepia* sp. FTIR analysis shows presence of amides, and spectra that resembled extant beta chitin. Finally, IHC reveals presence of molecules with specific reactivity to chitin anti-bodies indicating preserved organic material in *M*. *mississippiensis* is still recognizable as chitin.

Results of IHC support our interpretation of FTIR data as representative of chitin. Because spectra obtained in FTIR indicate chitin, the lack of a doublet in the Amide I region identifies the fossil material as beta chitin, which is also found in extant mollusks. Differences between spectra of fossil and extant chitin can be used to further illuminate changes in the β- chitin structure of the fossil.

β-chitin is characterized by parallel chains of chitin molecules held together with inter-chain hydrogen bonding [2]. The OH stretching absorbance (Figure 5B), at about 3445 cm^−1^ in extant chitin, is diminished in the fossil and shifted to lower wavenumbers (Table 2), showing that the specimen is losing OH by an as yet undetermined mechanism. The N-H asymmetric stretching vibration is shifted to slightly lower wavenumbers, showing that it is no longer hydrogen bonding exactly as in extant specimens. Changes in the region 2800–3600 cm^−1^ indicate that biomolecules have been degraded via disruption of interchain hydrogen bonds.

Examination of the region 800–1500 cm^−1^ shows that the spectrum of fossil material roughly follows the spectrum of the extant material (Figure 5D). This region has been referred to as the “crystallinity region” for oligosaccharides [23]. Loss of well-defined peaks in the fossil material is ascribed here as a loss of long-range ordering in strands of chitin due to shortening of the chitin strands. Similar changes in FTIR spectra have been observed in the breakdown of chitosan [24] and the organic components of nacre [25].

**Figure 8:**
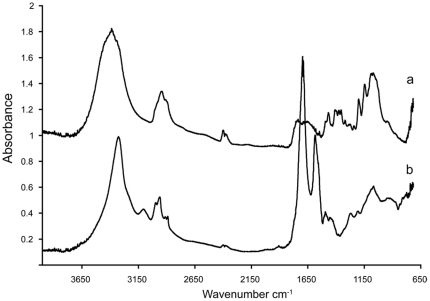
Comparison of two FTIR spectra of *M*. *mississippiensis* (MGS 1951). a) spectrum lacks Amide I and Amide II peaks, b) for comparison, spectrum shows the Amide I peak at 1656 cm^−1^ and Amide II peak at 1545 cm^−1^.

Shortening of strands and loss of hydrogen bonds could explain morphological differences between de-mineralized *M*. *mississipiensis* and extant *Sepia* sp. seen in Hyperprobe (Figure 3A,B) and IHC (Figure 6A,B). In addition, not all pieces of de-mineralized fossil showed amide or lipid peaks (Figure 8). Differences observed in fossil chitin, as compared to extant, could be a result of mechanical destruction, preservational biases or partial chemical degradation within microenvironments of the specimen.

There is a remote possibility that the organics detected in the fossil are a result of contamination. This is highly unlikely. Chitin is not a common lab contaminant. Fossil specimens used in this study were surface collected, washed in water and stored in climate controlled metal cabinets until they were sent to us in individual sealed plastic bags. None of the samples used for Hyperprobe, FTIR or IHC were preserved using epoxies or other glues. To reduce possible contaminants from extant samples chemical preparations were done in separate labs and FTIR was conducted in a mineralogy lab that has never housed invertebrate specimens. Still, there is a slight possibility that the chitin detected in our samples could a result of extant fungus. This also is unlikely, as we saw no evidence of fungi in SEM. FTIR results showed β-chitin in our samples not γ-chitin found in fungi [2]. Results of IHC were specific for chitin (Figure 7), thus it is unlikely that the amide peaks seen in FTIR (Figure 6, Table 1) are result of contaminating proteins from human or other sources.

### Mechanisms for preservation

In vivo inorganic-organic structure of the cuttlebone [13], in combination with physical and geochemical conditions within the depositional environment [26], [27] and favorable taphonomic factors likely contributed to preservation of organics in *M*. *mississippiensis*. Available clays within the Yazoo Clay in conjunction with suboxic depositional environment [28]–[30] may have facilitated preservation of original organics by forming a physical and geochemical barrier to degradation [31]–[33]. One key to the preservation of organic tissues, in particular chitin and chitosan, is cessation of bacterial degradation within environments of deposition. Bacterial breakdown of polymeric molecules is accomplished through activities of both free extracellular enzymes (those in the water column) and ektoenzymes (those on the surface of the microbial cell) such as chitinases [31], [32]. Chitinases function either by cleaving glycosidic bonds that bind repeating *N*-acetyl-D-glucosamine units within chitin molecules or by cleaving terminal *N*-acetyl-D-glucosamine groups [33]–[35]. These enzymes adsorb to the surface of clay particles, which inactivates them [33]–[35]. Strong ions in solution like iron may act in the same manner [33]. Once bound to functional groups within these polymeric molecules, Fe^2+^ ions prevent specific bond configuration on the active-site cleft of specific bacterial chitinases and prevents hydrolysis [31], [33], thus contributing to preservation.

Organic layers within cuttlebones are protected by mineralized layers, similar to collagen in bones, and this mineral-organic interaction may also have played a role in their preservation [36]–[38]. Specimens of *M*. *mississipiensis* show preserved original aragonite as well as apparent original organics. These organics appear to be endogenous and not a function of exogenous fungal or microbial activity. Fungi contain the γ form of chitin not the β allomorph found in our samples. Also, SEM analyses shows there is no evidence of tunneling, microbes, or wide-spread recrystallization of the aragonite. Therefore the chitin-like molecules detected in fossil sample are most likely endogenous. Similar to collagen in bone, perhaps, organics could not be attacked by enzymes or other molecules until some inorganic matrix had been removed [39].

### Conclusions

Analysis by Hyperprobe, FTIR and IHC of cuttlebones of the Eocene coleoid cephalopod *M*. *mississipiensis* show preservation of degraded original organics consistent with β-chitin. These are oldest known organics consistent with β-chitin (34.36 mya) [8] and are the first from a fossil marine mollusk. Preservation of organics consistent with β-chitin was possible due to the inorganic/organic construction of cuttlebone and available free Fe^2+^ ions in suboxic depositional environment, which inhibited microbial or enzymatic degradation. Future work will focus on comparisons with other Eocene cuttlefish and the phylogenetic implications of chitinous structures with regards to the origin cuttlefish.

## Supporting Information

Text S1 **Supplementary text.**
(DOC)
