# Supplementary material for: Characterization of Organics Consistent with β-Chitin Preserved in the Late Eocene Cuttlefish Mississaepia mississippiensis
Source: PLoS One. 2011 Nov 23;6(11):e28195. doi: 10.1371/journal.pone.0028195 (PMC3223232; doi:10.1371/journal.pone.0028195)
Supplement: Text S1 — Supplementary text. (DOC) [file pone.0028195.s001.doc]

**Text S1. Supplementary text.**

**SEM/EDS:**

Fossil specimens were cut and polished, coated in gold and were analyzed using Hitachi S-4 300 SEM and energy dispersive spectrometer (EDS). Chemical analyses were performed at accelerating voltage 15 kV and energy calibration was measured on standard minerals. All elements were analyzed and no peaks were omitted.

**De-mineralization:**

Demineralization was preformed using 0.5 M HCl for all samples. Samples of extant *Sepia* sp. (NCSM 11402 and 11403) were powdered using a sterilized mortar and pestle. Approximately 49.90 mg to 55.94 mg of powdered cuttlebone was placed in 50 ml vials containing 5 ml of 0.5 M HCl. After three hours the powdered cuttlebone was centrifuged for 15 minutes at 10391 RCF (Relative Centrifugal Force).

Gladii of three extant *Loligo* sp. were crushed to approximately 1 x 5 mm pieces using a sterilized mortar and pestle. Approximately 9.9 mg to 11.1 mg of sample were placed in 2 ml vials containing 1ml of 0.5 M HCl. Samples were removed from the solutions after 1, 2, 4, and 24 hours.

Extinct coleoid (*M*. *mississippiensis* MGS 1951) was powdered using a sterilized mortar and pestle. Approximately 750.7mg of powder was placed in a 50 ml vial containing 10 ml of 0.5 M HCl. After 72 hours, the sample was centrifuged at 10391 RCF, the soluble fraction was removed and saved. The remaining insoluble fraction was transferred to seven 2ml vials for further centrifuging and washing at 16000 RCF.

All pellets were then washed multiple times in order to remove all remaining HCl, frozen in a -80°C freezer and lyophilized with a FreeZone 18 Liter Freeze Dry System.

**Hyperprobe:**

De-mineralized powders of extant *Sepia* sp. (NCSM 11403 &NCSM 11402) and of fossil *M. mississippiensis* (MGS 1951) were mounted to separate glass slides using carbon tape. These specimens were then carbon coated using a Denton vacuum BTT-IV to a carbon thickness of ~150 Angstroms. Specimens were then analyzed using a Jeol X-ray Analyzer JXA-85-30F at 10kV and 3,000x for both samples. The spectrometer crystal (LDE) was set on the nitrogen peak using boron nitride as the standard. When the LDE registered a count, a dot was placed on the image at the point of the beam.

**FTIR:**

De-mineralized and mineralized samples were analyzed from 600-4000 cm^-1^ using a Nicolet 6700 FTIR bench from Thermo Scientific, in conjunction with a Continuum IR microscope with 15x objective. Samples were placed on the top of a 13x2mm KBr plate that had been commercially pre-prepared. Resolution was 1 cm^-1^. Unpolarized radiation was used, but earlier evaluation shows that the “unpolarized” light was partially polarized by reflection on internal mirrors such that min/max absorbance of highly polarized bands for apatite was 0.76. This wasn’t deemed significant in the study presented here.

**IHC:**

De-mineralized samples of extant *Sepia* sp. were fixed in 10% neutral buffered formalin for 1hr at RT, and washed 2 times for 5 minutes in PBS buffer. The demineralized fossil *M*. *mississippiensis* and fixed modern sections were dehydrated with two changes of 70% ethanol, infiltrated with L.R. White and 70% ethanol (2:1) mixture, with two changes of pure LR white, placed specimen in bottom of Gelatin capsules and filled to the brim with L.R. White, then capped with the other half of the capsule. Capsules were then polymerized in 60^o^C oven for 24-48 hours and cut into 200 µm ultra thin sections using a Leica EM UC6 microtome. Sections were transfered to 6 well "PTFE" Printed Slides and set, covered, on a 45^o^C hot plate for initial drying, then were placed in a 45^o^ C drying oven overnight. Chitinase (Streptomyces grisus) purchased from Sigma Aldrich (Product number C6137) was mixed at 1mg/ml ratio with a 50mM pH 6.0 buffered potassium phosphate solution. Sections (in the wells designated for chitinase analysis) of both fossil and extant samples were then inoculated with 100ul of chitinase solution per well at 25^o^C one change per hour for 7 hours then left overnight. Wells that contained the chitinase solution were washed three times with TBS for five minutes each time to remove any left over chitinase solution. To retrieve the antigen or to unmask the epitopes through the removal of calcium ions, all wells were then incubated twice with 0.5 M EDTA pH 8.0 for ten minutes each time. All wells were then re-washed three times in (Tris Buffered Solution) TBS for five minutes each time to remove any leftover EDTA. To quench auto-fluorescence, and to unmask any epitopes that may have been blocked by cross-linking or other artifact, all wells were then incubated twice with sodium borohydride (NaBH_4_) 1mg/ml in E-pure water for ten minutes. All wells were then re-washed three times in TBS for five minutes each time to remove any leftover NaBH_4_ solution. To block nonspecific binding of antibodies, both primary and secondary, all wells were then incubated for one hour in 1% (Bovine Serum Albumen) BSA in TBS with 1% goat serum (blocking buffer). Rbt anti-chitin antibodies developed at the University of Ulm, Germany [18] were put into solution at a dilution of 1 to 60 (13.3ul Rbt anti-chitin antibody to 800ul blocking buffer (1% BSA in TBS with 1% goat serum)). Chitin antibody solution was added to the wells that had been subjected to the chitinase solution and to other wells (both fossil and modern) designated for use as the positive. To show that antibody binding is specific to chitin, Rbt anti-human testosterone, an irrelevant antibody, solution at a dilution of 1 to 60 (13.3ul Rbt anti-human testosterone to 800ul blocking buffer (1% BSA in TBS with 1% goat serum)) was added to wells designated for negative control (both fossil and modern). Blocking buffer was added to wells designated for secondary antibody only and all were set in a 4^o^C refrigerator overnight., then all wells were washed in TBS three times for 5 minutes each time, to remove any leftover antibodies. Afterwards, secondary antibody solution (Biotinylated Goat Anti-Rabbit IgG (H+L) at 1:500 in the blocking buffer (8ul secondary antibody to 4ml blocking buffer (1% BSA in TBS with 1% goat serum)), was added to all wells and let set for 1 hour at room temperature.. Subsequently, all wells were washed three times in TBS for five minutes each time, to remove any leftover secondary antibodies. Fluorescein Avidin D (from Vector A2001) was mixed at 1:1000 in TBS (4ul Fluorescein Avidin D to 4 ml TBS ). Fluorescein Avidin D solution was added to all wells and left in the dark for 1 hour. Fluorescein Avidin D binds to the bound primary/secondary antibodies. All wells were then washed three times in TBS for five minutes each time, to remove any leftover Fluorescein solution. TBS solution was then removed and slides were partially dried in air. Slides were then mounted with coverslips using Vectasheild H-1000 mounting medium for fluorescence. Fluorescence was read using an Axio Cam MRc5 on a ZEISS Axioskop 2 plus microscope at 63X, 35ms with same brightness and contrast for all wells and processed with the computer program AxioVision 4.7.0.0.
